# Correction: Rare disease in Malaysia: Challenges and solutions

**DOI:** 10.1371/journal.pone.0273939

**Published:** 2022-08-26

**Authors:** Asrul Akmal Shafie, Azuwana Supian, Mohamed Azmi Ahmad Hassali, Lock-Hock Ngu, Meow-Keong Thong, Hatijah Ayob, Nathorn Chaiyakunapruk

In the Rare disease awareness and patient advocacy subsection of the Results, there is an error in the second sentence of the third paragraph. The correct sentence is: Additionally, there are also many other societies, such as the Welfare Association for Dwarfism Malaysia and Spinal Muscular Atrophy (SMA) Malaysia, that represent patients according to the specific disorder.

## References

[1] Shafie AA, Supian A, Ahmad Hassali MA, Ngu L-H, Thong M-K, Ayob H, et al. (2020) Rare disease in Malaysia: Challenges and solutions. PLoS ONE 15(4): e0230850. 10.1371/journal.pone.0230850 32240232 PMC7117672

