# Supplementary material for: Rare disease in Malaysia: Challenges and solutions
Source: PLoS One. 2020 Apr 2;15(4):e0230850. doi: 10.1371/journal.pone.0230850 (PMC7117672; doi:10.1371/journal.pone.0230850)
Supplement: S1 File — (DOCX) [file pone.0230850.s002.docx]

**Supporting Information: Interview Guides**

Semi-structured interview guide for:

1. Healthcare Providers: The Pharmaceutical Services Division (PSD) &
   The National Pharmaceutical Regulatory Agency (NPRA)
2. Healthcare Providers: Consultants / Specialists
3. Society & Volunteers
4. Pharmaceutical Industries

Appendix 3

Semi-structured interview guide for

ID No:

**HEALTHCARE PROVIDERS**

PHARMACEUTICAL SERVICES DIVISION (PSD) &
NATIONAL PHARMACEUTICAL CONTROL BUREAU (NPCB)

**DEVELOPMENT OF ORPHAN DRUGS COVERAGE DECISION FRAMEWORK IN MALAYSIA**

*My name is* ***Azuwana Supian,*** *and I am a PhD candidate of Universiti Sains Malaysia. My interests in this research are the issues of rare disease and orphan drug in Malaysia, how we can ensure adequate, continuous, and equitable access to quality, safety, effectiveness, and affordable medicines towards achieving optimal health outcomes. Maybe you can say or suggest something that can help the policymakers to consider new system or guideline of orphan drug in Malaysia.*

*I’m interested in hearing what you have to say about this issue.*

*To begin with, could you please tell me something about yourself?*

| **Demographic data** | | |
| --- | --- | --- |
| Age |  | Gender: M F |
| Agency/Facility |  | |
| Position |  | |
| Years in service |  | |

| **Section 1: Policy and system** | |
| --- | --- |
| 1. | Would you please describe the most important factors to consider in the decision to register/listing orphan drug? |
| 2. | What are the barriers present for a new orphan drug to enter Malaysia market? |
| 3. | How does the government facilitate the designation and registration of orphan drugs?  Is there any particular guideline or procedure for an orphan drug to register/ listing in Malaysia? |
| 4. | Can you name the countries for Malaysia as a reference in orphan drug designation and registration? |

| **Section 2: Personnel and Product** | |
| --- | --- |
| 1. | What is the role of the panel/ peer-review reviewers?  Do these panels contain rare disease experts? |
| 2. | What are the most important factors to consider in the decision to register / listing orphan? |
| 3. | [Can a product already authorise for a non-orphan indication in Malaysia receive the orphan designation for another indication which is orphan?](http://www.ema.europa.eu/ema/index.jsp?curl=pages/regulation/q_and_a/q_and_a_detail_000014.jsp&mid=WC0b01ac058061ecb9) |
| 4. | How about if a medicinal product has already granted orphan drug designation in other countries, would this be automatically accepted for Malaysia? |
| 5. | What are the incentives available for orphan drugs in Malaysia? |
| 6. | Do you think that public fund and major charitable funding bodies make enough funding available for rare disease and orphan drug? |

***Thank you for your time and participation!***

Semi-structured interview guide for

ID No:

**HEALTHCARE PROVIDERS**

CONSULTANTS/SPECIALISTS

**DEVELOPMENT OF ORPHAN DRUGS COVERAGE DECISION FRAMEWORK IN MALAYSIA**

*My name is* ***Azuwana Supian,*** *and I am a PhD candidate of Universiti Sains Malaysia. My interests in this research are the issues of rare disease and orphan drug in Malaysia. How we can ensure adequate, continuous and equitable access to quality, safety, effectively and affordable medicines towards achieving optimal health outcomes. Maybe you can say or suggest something that can help the policymakers to consider new system or guideline of orphan drug in Malaysia.*

*I’m interested in hearing what you have to say about this issue.*

*To begin with, could you please tell me something about yourself?*

| **Demographic data** | | |
| --- | --- | --- |
| Age |  | Gender: M F |
| Agency/Facility |  | |
| Position |  | |
| Years in service |  | |

| **Section 1: Awareness, understanding, attitudes & exposure** | |
| --- | --- |
| 1. | Would you please describe the views of Malaysian physicians on the orphan drug? |
| 2. | What programs are available for Malaysian physicians’ education and awareness on the orphan drug? |
| 3. | How would you describe patients’ acceptance of rare disease in Malaysia? |
| 4. | What programs are available for patients’ education and awareness of rare disease? |

| **Section 2: Opinion in rare disease & treatment (orphan drug)** | |
| --- | --- |
| 1. | What strategies could be used to improve orphan drug access in Malaysia? |
| 2. | What are the biggest challenges for orphan drug? |
| 3. | What barriers are present to the entry of a new orphan drug in Malaysia? |
| 4. | What do you think will be the most significant trend or change to the orphan and rare disease sector between now and 2020? |
| 5. | Can you suggest the reference country for Malaysia to follow in rare disease and orphan drug system? |
| 6. | Do you think that public fund and major charitable funding bodies make enough funding available for rare disease and orphan drug? |

***Thank you for your time and participation!***

ID No:

Semi-structured interview guide for

**SOCIETY & VOLUNTEER**

**DEVELOPMENT OF ORPHAN DRUGS COVERAGE DECISION FRAMEWORK IN MALAYSIA**

*My name is* ***Azuwana Supian,*** *and I am a PhD candidate of Universiti Sains Malaysia. My interests in this research are the issues of rare disease and orphan drug in Malaysia. How we can ensure adequate, continuous and equitable access to quality, safety, effectively and affordable medicines towards achieving optimal health outcomes. Maybe you can say or suggest something that can help the policymakers to consider new system or guideline of orphan drug in Malaysia.*

*I’m interested in hearing what you have to say about this issue.*

*To begin with, I was wondering if you could tell me something about yourself:*

| **Demographic data** | | |
| --- | --- | --- |
| Age |  | Gender: M F |
| Occupation |  | Salary/income: RM ………………….. / month |
| Name of society/volunteer |  | |
| Years in membership |  | |

| **Section 1:** **Issues & problems** | |
| --- | --- |
| 1. | Could you describe the health care system of rare disease and its treatment in Malaysia? |
| 2. | How can patients or public assess the information of rare disease? |
| 3. | What is the situation of information on rare diseases in general? |

| **Section 2:** **Organisation** | |
| --- | --- |
| 1. | Would you please tell more about your organisation and its operations?   - Date started & location - Mission / goals - Main funding & sources - Major achievements |
| 2. | How can people assess the information about this organisation and be a member? |
| 3. | So far, what has been this main organisation problem? |
| 4. | What have been your principal problems in this organisation? |

| **Section 3:** **Willingness to pay** | |
| --- | --- |
| 1. | What do you think about public and major charitable funding bodies?  Is it enough funding available for rare disease treatment? |
| 2. | How much do you willing to pay for cost or co-payment of rare disease treatment? |
| 3. | What do you hope to achieve in the healthcare system in Malaysia for rare disease patients? |

***Thank you for your time and participation!***

ID No:

Semi-structured interview guide for

**PHARMACEUTICAL INDUSTRIES**

**DEVELOPMENT OF ORPHAN DRUGS COVERAGE DECISION FRAMEWORK IN MALAYSIA**

*My name is* ***Azuwana Supian,*** *and I am a PhD candidate of Universiti Sains Malaysia. My interests in this research are the issues of rare disease and orphan drug in Malaysia. How we can ensure adequate, continuous and equitable access to quality, safety, effectively and affordable medicines towards achieving optimal health outcomes. Maybe you can say or suggest something that can help the policymakers to consider new system or guideline of orphan drug in Malaysia.*

*I’m interested in hearing what you have to say about this issue.*

*To begin with, I was wondering if you could tell me something about yourself:*

| **Demographic data** | | |
| --- | --- | --- |
| Age |  | Gender: M F |
| Agency |  | |
| Position |  | |
| Years in the industry |  | |

| **Section 1: Registration of Orphan Drug** | |
| --- | --- |
| 1. | How does the government facilitate registration of orphan drugs?  Is there any particular guideline or procedure for orphan drug to register/ listing in Malaysia? |
| 2. | What is the fee of registration and average time of marketing approval of orphan drugs? |
| 3. | In your opinion, how transparent the process of approval for an orphan drug in Malaysia? |
| 4. | How about if a medicinal product has already granted orphan drug designation in other countries, would this be automatically accepted for Malaysia? |
| 5. | Can an application for orphan medicinal product designation be submitted at any time in the development process? |
| 6. | Do you agree that the Malaysian Commission should propose legislation to regulate rare disease patient registers across Malaysia? |
| 7. | Can you suggest the reference country for Malaysia to follow in orphan drug designation and registration? |

| **Section 2:** **Product (Orphan Drug)** | |
| --- | --- |
| 1. | What are the incentives available for orphan drugs in Malaysia? |
| 2. | How would you describe the market exclusivity (patent) system of orphan drugs? |
| 3. | What barriers are present to the entry of a new orphan drug in Malaysia? |
| 4. | What do you think will be the most significant trend or change to the orphan and rare disease sector between now and 2020? |

| **Section 3: Fund and cost** | |
| --- | --- |
| 1. | May I know, who and how prices setting for orphan drugs in Malaysia market? |
| 2 | Do you think that public fund and major charitable funding bodies make enough funding available for rare disease and orphan drug? |
| 3. | How your organisation can help those unfortunate patients? |
