# Supplementary material for: Rare disease in Malaysia: Challenges and solutions
Source: PLoS One. 2020 Apr 2;15(4):e0230850. doi: 10.1371/journal.pone.0230850 (PMC7117672; doi:10.1371/journal.pone.0230850)
Supplement: Attachment — Submitted filename: 2. Response to Reviewers (final).docx [file pone.0230850.s003.docx]

**Rare disease in Malaysia: Challenges and solutions**

**Response to Reviewers**

| 1. | Is the manuscript technically sound, and do the data support the conclusions?  The manuscript must describe a technically sound piece of scientific research with data that supports the conclusions. Experiments must have been conducted rigorously, with appropriate controls, replication, and sample sizes. The conclusions must be drawn appropriately based on the data presented.  Reviewer #1: Partly  Reviewer #2: Partly  **Response**  **Experiments (methodology) were described in protocols.oi**  DOI: dx.doi.org/10.17504/protocols.io.6zdhf26  <https://www.protocols.io/view/rare-disease-in-malaysia-6zdhf26> |
| --- | --- |
| 2. | Has the statistical analysis been performed appropriately and rigorously?  Reviewer #1: N/A  Reviewer #2: N/A  **Response: not applicable in this study** |
| 3. | Have the authors made all data underlying the findings in their manuscript fully available?  The [PLOS Data policy](http://www.plosone.org/static/policies.action#sharing) requires authors to make all data underlying the findings described in their manuscript fully available without restriction, with rare exception (please refer to the Data Availability Statement in the manuscript PDF file). The data should be provided as part of the manuscript or its supporting information, or deposited to a public repository. For example, in addition to summary statistics, the data points behind means, medians and variance measures should be available. If there are restrictions on publicly sharing data—e.g. participant privacy or use of data from a third party—those must be specified.  Reviewer #1: No  Reviewer #2: No  **Response :**  **Data Availability Statement: data can be access upon article acceptance** |
| 4. | Is the manuscript presented in an intelligible fashion and written in standard English?  PLOS ONE does not copyedit accepted manuscripts, so the language in submitted articles must be clear, correct, and unambiguous. Any typographical or grammatical errors should be corrected at revision, so please note any specific errors here.  Reviewer #1: No  Reviewer #2: Yes  **Response :**  **We have now send the manuscript to a different professional English proof reading service (American Journal Expert- the certificate as attached)** |

**Reviewer #1:**

| 1. | The information is outdated. It needs to be updated by revisiting the literature and redoing the interviews.  **Response :**  **The reference is now updated in the revised manuscript. Anothre four new references are added.** |
| --- | --- |
| 2. | The checklist for qualitative research studies has not been filled.  **Response :**  **The reference is now updated in the revised manuscript. Another four new references are added.** |
| 3. | It is difficult to assess how much data this paper has captured. It appears quite thin. The questionnaire needs to be included as a supplementary file. Also, provide detailed supplementary files of the interviews, as in the recent paper “The role of patient organizations in the rare disease ecosystem in India: An interview-based study.” Orphanet J Rare Dis. 14:117 (2019). Make a list of the literature which was consulted in the triangulation process, and include this as a supplementary file.  **Response :**  **The semi-structured interview guide is now included in supporting information (attachment)** |
| 4. | The English needs improvement. There are many awkward phrases, such as in Line 112, Line 108. Some lines are confusing such as Line 121 -Line 122. Even quotes should be edited for correct English.  **Response :**  **We have now send the manuscript to a different professional English proof reading service (American Journal Expert)** |

**Reviewer #2:**

In the manuscript, Shafie et al. report the current situation of and the recent advances in rare disease realm in Malaysia via cross-sectional study, document-survey and interviews. Authors gathered the information from the multiple points of view, including healthcare system and governance, clinical expertise and patient management, newborn screening, funding and rare disease awareness and patient advocacy, which has high importance in terms of strategic development of new healthcare policies and R&D programs in developing countries, however, the manuscript still holds major and minor issues of concern.

| 1. | The limitation of this cross-sectional study/analysis is its lack of estimation of the numbers of rare diseases/patients expanding toward the entire country, which lead to their underestimation. Rare disease patients could be seen and treated in the other general private and public hospitals than the Ministry of Health Malaysia’s facilities or university hospitals. In order to claim that “they (funding in Malaysia) are not sufficient to cover the high number of rare disease patients”, the estimation would be indispensable.  **Response :**  **There is currently no epidemiological estimates for rare disease prevalence in Malaysia. Hence, the motivation for the study, we agree that cross sectional study design has inherent internal validity to accurately estmate disease with very rare prevalence. However, rare disease in Malaysia is usually treated in specialized service (genetic clinic).**    **This study collected data from public health facilities as only one private facility provided the service for rare disease (genetic clinic).**  **Public health facilities:**   a. **The Ministry of Health hospitals** b. **Non- Ministry of Health hospitals (teaching hospitals from the Ministry of Education)**   **Although the prevalent is still underestimated as there are patients who are not yet diagnosed, we believed this is the best estimate for the country. We have now added a new section to our manuscript to discuss this limitation.** |
| --- | --- |
| 2. | The WHO definition of rare diseases has to be referenced. In Europe, some diseases are excluded from the rare disease definition, such as infection and drug hypersensitivities, which handling should be described in the manuscript. And also, authors should mention the diseases which prevalence is less than 0.65% of the population.  **Response :**  **The WHO definition of rare disease already stated in the result:**  *To date, there is no official definition of rare disease for Malaysia yet. Even though the Malaysian Rare Disorders Society (MRDS), a voluntary organisation defines rare disease as a disease that affects less than 1 per 4000 in the community [19], feedback from the MOH however informally used the WHO definition of rare disease, any disease that has a prevalence between 0.65 - 1% of the population, and the diagnosis and treatment are complicated* |
| 3. | The demographics of the respondents should be shown as well. Especially, the department which the respondents belong to could bias the number of diseases/patients in rare disease realms.  **Response :**  **Demographic of respondents added into the manuscript**  *A total of twenty-six respondents were successfully interviewed in this study. Seven respondents were policymakers (three officers from the Pharmacy Policy & Strategic Planning Division, two officers from the Pharmacy Practice & Development Division and two officers from the National Pharmaceutical Regulatory Agency). Another four respondents were from genetic clinic (three from the Ministry of Health and one from the a university hospital and 12 responders were from the pharmaceutical industry as licensed holders of few orphan drugs. Meanwhile, three responders from the advocacy group were the presidents of leading rare disease society in Malaysia (Malaysian Rare Disorders Society, Malaysia Lysosomal Diseases Association and Malaysia Metabolic Society). However, two respondents from genetic clinics did not respond, and one was on an extended leave.* |
| 4. | If space permits, the paper would benefit from the following points of view;  Comparison of newborn screening tests covered by UHC between Malaysia and the other country which has implemented UHC.  **Response :**  **Comparison between Malaysia and the other country which has implemented UHC already discussed in our previous publication:**  Shafie, A. A., Chaiyakunapruk, N., Supian, A., Lim, J., Zafra, M., & Hassali, M. A. (2016). State of rare disease management in Southeast Asia. Orphanet journal of rare diseases, 11(1), 107.  doi:10.1186/s13023-016-0460-9  <https://www.ncbi.nlm.nih.gov/pmc/articles/PMC4969672/> |
