# Supplementary material for: Rare disease in Malaysia: Challenges and solutions
Source: PLoS One. 2020 Apr 2;15(4):e0230850. doi: 10.1371/journal.pone.0230850 (PMC7117672; doi:10.1371/journal.pone.0230850)
Supplement: Attachment — Submitted filename: 2. Response to Reviewers 4.docx [file pone.0230850.s004.docx]

**Rare disease in Malaysia: Challenges and solutions**

**Response to Reviewers 2**

| Additional editor comments | I do think the revision does display lack of clarity on the issue of the definition of rare diseases in Malaysia. For example, in the Abstract, under results section, you state in lines 54 to 56  **Response:**  Change in the abstract and results  **Abstract:**  Malaysia has no official definition of rare disease yet. However, the government frequently uses the World Health Organisation’s (WHO) definition of a rare disease as "any disease that is prevalent in 0.65% to 1% of the population”. Currently, there are 13 rare disease specialists and a dozen medical doctors in genetic clinics around Malaysia. From the survey, 1,249 patients diagnosed with rare diseases in public hospitals. Only 60% received their medications or supplements, and the rest continued with symptomatic treatment.  **Results:**  To date, there is no official definition of rare disease in Malaysia. Although the Malaysian Rare Disorders Society (MRDS), a voluntary organisation, defines rare disease as a disease that affects less than 1 in 4,000 people in a community [20], the MOH has routinely used the WHO definition of rare disease, which is ‘any disease that is prevalent in 0.65% to 1% of the population, and the diagnosis and treatment are complicated’ in their operation [11]. However, the MOH currently in the process of reviewing the rare disease definition for Malaysia. |
| --- | --- |
| Issue 6 | As commented by Ed2, authors must review the “original” source (≠ref #11) of the WHO definition of rare diseases (White paper? Website?). From the original WHO source(s), authors would find the reason why WHO set “0.65-1.00%” for the prevalence of rare disease definition, and should provide a considerable explanation about the reason why authors excluded the diseases which prevalence is less than 0.65% of the population from this survey. In order to promote creation of a clear rare disease national plan in Malaysia, solution(s) should be considered not only for the “relatively common” rare disease patients but also for people living with rarer diseases.  **Response:**  We agreed that the review of the original source could shed light on the genesis of the definition. In this study, feedback from the Ministry of Health Malaysia indicates that there is no official definition of rare disease in Malaysia yet. However, the ministry provides us with definition that they have informally adopted i.e*. “any disease that is prevalent in 0.65% to 1% of the population, and the diagnosis and treatment are complicated*”. This is purportedly based on the definition by the World Health Organisation (WHO). The ministry also attached the list of rare diseases together in their feedback. Usually, we do not argue the decision and policy statement from the ministry.  <refer to the attachment, it is not to be published as it is confidential>  Unfortunately, we failed to find the original sources of WHO definition but only a sentence from Song et.al (2012) in their introduction.  Song, P., et al. (2012). "Rare diseases, orphan drugs, and their regulation in Asia: Current status and future perspectives." Intractable Rare Dis Res **1**(1): 3-9.   |
