# Supplementary material for: Rare disease in Malaysia: Challenges and solutions
Source: PLoS One. 2020 Apr 2;15(4):e0230850. doi: 10.1371/journal.pone.0230850 (PMC7117672; doi:10.1371/journal.pone.0230850)
Supplement: Attachment — Submitted filename: 2. RESPONSE TO REVIEWER V3.docx [file pone.0230850.s005.docx]

**Rare disease in Malaysia: Challenges and solutions**

**Response to Reviewers 3**

**Comment from the reviewer:**

I read your reference 11, and it's references that can reasonably be supposed to support the WHO definition stated in your manuscript, but could not find a reference to any WHO document. I searched the WHO site and did not find a section or document on their website that gave any definition. I did find a document that gave a definition adopted by the  Europe Union, which defines a rare disease as one that affects less than 0.1% 'of the population', which presumably refers to its prevalence.

I suggest that the authors conduct a search to back up the assertion in reference 11, that seems to have no clear backing. If they can find a reference, they should give it instead of, or in addition, to reference 11, and discuss why disease below the lower bound of that definition are omitted from consideration in the manuscript.

**Response:**

We agreed that reviewing the original source could shed light on the genesis of the definition. We did search for various WHO documents and publications when writing the manuscript. However, we cannot find any statement to support feedback from the Ministry of Health Malaysia which defines rare disease "any disease that is prevalent in 0.65% to 1% of the population”.

We suspect that this could be the case of ‘folklore’ definition whereby an ‘unofficial’ statement is adopted by the community and ministry. This does occasionally happens in the health sector. Usually, we do not argue the decision and policy statement from the ministry especially when it is made official by them. However, without strong evidence, the practice could pose a substantial challenge to future planning and discourse.

For the purpose of this manuscript and publication, we agreed to change in the abstract, results and discussion part, and also delete the reference number #11.

Below are the amendments to the manuscript:

Amendment 1

**Abstract:**

Malaysia has no official public definition of rare disease yet but currently in the process of reviewing them for Malaysia. There are 13 rare disease specialists and a dozen medical doctors in genetic clinics around Malaysia, mainly in public health facilities. From the survey, 1,249 patients diagnosed with rare diseases in public hospitals. Only 60% received their medications or supplements, and the rest continued with symptomatic treatment.

Amendment 2

**Results:**

To date, there is no official public definition of rare disease in Malaysia, even though the most prominent rare disease voluntary organisation called the Malaysian Rare Disorders Society (MRDS) defines rare disease as a disease that affects less than 1 in 4,000 people in a community [20]. However, upon request, the ministry provides us with a definition that they have internally adopted i.e*. “any disease that is prevalent in 0.65% to 1% of the population, and the diagnosis and treatment are complicated*” purportedly from the World Health Organisation (WHO). Despite the reference, we are unable to trace the WHO documents and publications that could support the definition. Currently, the MOH still in the process of reviewing the rare disease definition for Malaysia.

Amendment 3

# Discussion

In the management of rare diseases, the definition is fundamental to kick off further actions. Each country has its definition of rare diseases according to its prevalence, and it varies across regions. For example, in the USA, a disease is rare when it affects fewer than 200,000 people [1]. In the European Union, a disease is considered a rare disease when it affects less than one in 2,000 people [2]. Whereas in East Asia, Taiwan defines a rare disease if it occurs fewer than one in10,000 population [3]. The majority of the countries defined rare diseases as to be between 0.01% to 0.05% of their population. These percentages are far below the one used by the Malaysia MOH (*0.65% to 1%).* However, there are doubts on *the definition used by the Malaysian MOH given that there is no reference that could support it - possibly a ‘folklore’ definition’.* The definition of rare disease cannot be arbitrary as it could have a wrong impression, create misunderstanding and complicate discussion among rare disease stakeholders, especially the patients and caregivers. Nevertheless, the MOH now reviewing the country's rare disease definition to be in line with other reference countries as well as neighbouring countries, which will encourage cooperation in matters related to the management of rare diseases in the future.

In 2016, Helen Clark, the former administrator of the United Nations Development Programme stated at the 11th Annual International Conferences on Rare Diseases and Orphan Drugs that, “No country can claim to have achieved UHC if it has not adequately and equitably met the needs of those with rare diseases” [4]. This statement is very relevant to countries that implement total universal health coverage, such as Malaysia. The basic principle of UHC is that all diseases should be treated with readily available and essential treatments. Teerawattananon et al. (2016) suggested four building blocks for setting the effective priority of UHC [5]:

1. Governing structure with precise functions and regulation of institutes and their inter-relation;
2. Capacity building programmes by policymakers, researchers, and other stakeholders, including the general public for a better understanding of health priority;
3. Resource availability and mobilisation to support priority setting; and
4. Collaboration with networks of local, international, and global organisations that aim to support UHC policies.

…………………………………………..

……………………………………………….

…..

…..

1.	Senate and House of Representatives of the United States of America, *Rare Diseases Act of 2002*, N.I.o.H. USA, Editor. 2002: USA.

2.	The European Parliament and of The Council of the European Union, *Regulation (EC) No 141/2000 of The European Parliament and of The Council of 16 December 1999 on orphan medicinal products.* Journal of the European Communities, 2000: p. 1-5.

3.	Song, P., et al., *Rare diseases, orphan drugs, and their regulation in Asia: Current status and future perspectives.* Intractable Rare Dis Res, 2012. **1**(1): p. 3-9.

4.	United Nations Development Programme. *Helen Clark: Written statement at the 11th annual International Conference on Rare Diseases and Orphan Drugs*. 2016 [cited 2017 11 January]; Available from: <http://www.undp.org/content/undp/en/home/presscenter/speeches/2016/10/20/helen-clark-written-statement-at-the-11th-annual-international-conference-on-rare-diseases-and-orphan-drugs.html>.

5.	Teerawattananon, Y., et al., *Role of priority setting in implementing universal health coverage.* BMJ 2016. **532**: p. i244.
